# Baseline characteristics of participants in the LANDMARC trial: A 3‐year, *pan*‐india, prospective, longitudinal study to assess management and real‐world outcomes of diabetes mellitus

**DOI:** 10.1002/edm2.231

**Published:** 2021-02-08

**Authors:** Ashok K. Das, Ambrish Mithal, Shashank Joshi, K. M. Prasanna Kumar, Sanjay Kalra, A. G. Unnikrishnan, Hemant Thacker, Bipin Sethi, Romik Ghosh, Vaishali Kanade, Arjun Nair, Senthilnathan Mohanasundaram, Shalini K. Menon, Deepa Chodankar, Vaibhav Salvi, Chirag Trivedi, Godhuli Chatterjee, Subhankar Chowdhury, Nadeem Rais, Subhash K. Wangnoo, Abdul H. Zargar

**Affiliations:** ^1^ Pondicherry Institute of Medical Sciences (PIMS Puducherry India; ^2^ Medanta ‐ The Medicity Gurgaon India; ^3^ Lilavati Hospital Mumbai India; ^4^ Centre for Diabetes and Endocrine Care Bengaluru India; ^5^ Bharti Hospital Karnal India; ^6^ Chellaram Diabetes Institute Pune India; ^7^ Bhatia Hospital Mumbai India; ^8^ Care Hospitals Hyderabad India; ^9^ Sanofi India Ltd Mumbai India; ^10^ Sanofi‐Synthelabo India Ltd Mumbai India; ^11^ IPGME & R and SSKM Hospital Kolkata India; ^12^ Chowpatti Medical Centre Mumbai India; ^13^ Apollo Hospital Education and Research Foundation New Delhi India; ^14^ Center for Diabetes & Endocrine Care Srinagar India

**Keywords:** baseline characteristics, diabetes, diabetes management, diabetic complications, India

## Abstract

**Introduction:**

Longitudinal data on progression, complications, and management of type 2 diabetes mellitus (T2DM) across India are scarce. LANDMARC (CTRI/2017/05/008452), the first *pan*‐India, longitudinal, prospective, observational study, aims to understand the management and real‐world outcomes of T2DM over 3 years.

**Methods:**

Adults (≥25 to ≤60 years old at T2DM diagnosis; diabetes duration ≥2 years at enrollment; controlled/uncontrolled on ≥2 anti‐diabetic agents) were enrolled. Baseline characteristics were analyzed using descriptive statistics.

**Results:**

Of the 6279 recruited participants, 6236 were eligible for baseline assessment (56.6% [*n*/*N* = 3528/6236] men; mean ± SD age: 52.1 ± 9.2 years, diabetes duration: 8.6 ± 5.6 years). mean ± SD HbA1c, fasting plasma glucose, and postprandial glucose values were 64 ± 17 mmol/mol (8.1 ± 1.6%), 142.8 ± 50.4 mg/dl, and 205.7 ± 72.3 mg/dl, respectively. Only 25.1% (*n*/*N* = 1122/6236) participants had controlled glycemia (HbA1c < 53 mmol/mol, <7%). Macrovascular and microvascular complications were prevalent in 2.3% (*n*/*N* = 145/6236) and 14.5% (*n*/*N* = 902/6236) participants, respectively. Among those with complications, non‐fatal myocardial infarction (*n*/*N* = 74/145, 51.0%) and neuropathy (*n*/*N* = 737/902, 81.7%) were the most reported macrovascular and microvascular complication, respectively. Hypertension (*n*/*N* = 2566/3281, 78.2%) and dyslipidemia (*n*/*N* = 1635/3281, 49.8%) were the most reported cardiovascular risks. Majority (74.5%; *n*/*N* = 4643/6236) were taking oral anti‐diabetic drugs (OADs) only, while 24.4% (*n*/*N* = 1522/6236) participants were taking OADs+insulin. Biguanides (*n*/*N* = 5796/6236, 92.9%) and sulfonylureas (*n*/*N* = 4757/6236, 76.3%) were the most reported OADs. Basal (*n*/*N* = 837/6236, 13.4%) and premix (*n*/*N* = 684/6236, 11.0%) insulins were the most reported insulins.

**Conclusions:**

Baseline data from LANDMARC help understand the clinical/medical profile of study participants and underscore the extent of suboptimal glycemic control and prevalence of associated complications in a vast majority of Indians with T2DM.

## Graphical abstract

People with long‐standing diabetes are at an increased risk of diabetes complications and cardiovascular events. In India, progression of diabetes and its complications over a long time has not been studied extensively. The LongitudinAl Nationwide stuDy on Management And Real‐world outComes of diabetes (LANDMARC) study has enrolled 6279 people with type 2 diabetes across India and will prospectively gather data on disease control, treatment, concomitant complications, and risks in these participants over 3

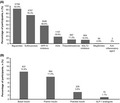

**What’s new**

- People with long‐standing diabetes are at an increased risk of diabetes complications and cardiovascular events. In India, progression of diabetes and its complications over a long time has not been studied extensively.
- The LongitudinAl Nationwide stuDy on Management And Real‐world outComes of diabetes (LANDMARC) study has recruited 6279 people with type 2 diabetes across India and will prospectively gather data on disease control, treatment, concomitant complications and risks in these participants over 3 years. LANDMARC baseline data represent the real‐world snapshot of a type 2 diabetic profile and underscore poor glycemic control and a considerable burden of complications prevalent in India.

**Previous presentation**

Part of the data in this article was presented at 79th Scientific Sessions of American Diabetes Association 2019, California (Poster number 1551‐P) and at International Diabetes Federation Congress 2019, Busan (Abstract Number: BU‐02934; Poster number P‐0553).

## 1 INTRODUCTION

A sharp increase in type 2 diabetes mellitus (T2DM) cases is predicted globally in the years to come. In 2019, an estimated 463 million adults worldwide (aged 20–79 years) had diabetes; this number is expected to increase to 700 million by 2045.^1^ In Southeast Asia, in 2019, there were ~87.6 million adults (aged 20–79 years) with diabetes, imparting a regional prevalence of 8.8%. This number is projected to increase to 153 million by 2045.^2^, ^3^ India, in 2019, had over 77 million cases of diabetes, which was the second highest in the world after China (116.4 million cases).^4^

Diabetes, a complex long‐standing disease, demands unending medical care and self‐management knowledge to avert or reduce acute and chronic complications.^5^ However, several issues related to sociocultural beliefs, people's attitude, physician barriers, treatment inertia, access to an adequate healthcare system, and financial constraints hinder optimal management of diabetes.^6^ In India, approximately 75% of people with diabetes are lost at each step of the diabetes care cascade (awareness stage [47.0%], treatment stage [12.0%], and failure to achieve control despite treatment stage [16.0%]), as indicated by the fourth National Family Health Survey (NFHS‐4)—a population‐based household survey—involving 729,829 participants (those with diabetes: 19,453 participants) aged 15–49 years from all states and territories of India.^7^ Consequentially, there is marked diabetes‐related morbidity and mortality. In 2019, Southeast Asia had ~1.2 million diabetes‐related deaths, and ~1 million of these diabetes‐related deaths were in India (age group: 20–79 years).^3^, ^8^

Real‐world data on the current continuum of diabetes care can provide valuable insights required for designing efficient management strategies and assessing healthcare system performance in India. Although the incidence and prevalence of diabetes has and is being studied judiciously through cross‐sectional studies,^9^, ^10^, ^11^, ^12^, ^13^ longitudinal data on understanding the development of diabetic complications over a period of time and their regional occurrences and outcomes are scarce or non‐existent in India.

The LongitudinAl Nationwide stuDy on Management And Real‐world outComes of diabetes (LANDMARC) is being conducted to investigate the incidence of macrovascular and microvascular complications, assess glycemic control, and evaluate treatment adaptation over a period of 3 years in participants with T2DM across India. The aim of the present analysis was to describe the baseline data of participants enrolled in the LANDMARC study.

## 2 PARTICIPANTS AND METHODOLOGY

The details of the design and methodology of the LANDMARC study have been published earlier^14^ and are briefly summarized in following sections.

### 2.1 Study design and participants

LANDMARC is the first national, multicenter, longitudinal, prospective, observational real‐world study to investigate a large cohort of people with T2DM across India over a period of 3 years. Adults ≥25 years and ≤60 years of age at the time of diagnosis, having T2DM for a duration of ≥2 years and controlled/uncontrolled on ≥2 anti‐diabetic agents, were recruited. People with known type 1 diabetes mellitus and secondary diabetes (e.g., gestational diabetes and fibrocalculus pancreatic diabetes) having limited life expectancy due to terminal diseases, and those on an investigational drug or those who had participated in a clinical trial in the previous 3 months, were excluded.

The study was approved by the Ethics Committee of the participating sites. The protocol complies with the Declaration of Helsinki, the principles laid by the 18th World Medical Assembly (Helsinki, 1964) and all subsequent amendments. The study is also aligned with the guidelines for Good Epidemiology Practice (US & European) and the local regulations, ethics committees (institutional review board/independent ethics committee), and applicable authorities. All participants provided signed informed consent before study participation and data collection/ documentation.

### 2.2 Selection of investigators

Investigators (general practitioners, endocrinologists, and diabetologists) willing to participate in the study were selected based on requisite qualification and availability of resources to conduct this study. Approximately 450 sites were planned to represent India across regions (East, West, North, and South), urban/semi‐urban practices, clinic/hospital bases, and government/corporate hospitals/nursing homes.

### 2.3 Data collection

Data are being recorded prospectively during the follow‐up at the end of every six months: 6 months (±14 days), 12 months (±25 days), 18 months (±30 days), 24 months (±30 days), 30 months (±40 days), and 36 months (±45 days). The study will complete at the end of 36 months (±45 days).

Data are being collected in electronic‐case report forms (e‐CRF). The data collected at baseline visit include the following: informed consent, eligibility criteria check, demographic characteristics, anthropometry, diabetes medical history, information related to known diabetes complications (myocardial infarction, stroke, peripheral vascular disease, neuropathy, nephropathy, retinopathy, acute coronary syndrome, heart failure, and unstable angina), and known cardiovascular (CV) risks (hypertension, dyslipidemia, and albuminuria). Details such as family history of premature coronary disease (PCD, a CV risk); laboratory test values for fasting plasma glucose (FPG), postprandial glucose (PPG) and glycated hemoglobin (HbA1c) and the class of oral and injectable anti‐diabetic drugs received by the participants were documented. A participant tracking log was used to record data (such as participant number, enrollment status: Yes or No, and reason for non‐enrollment in the study) in line with the data privacy requirement.

### 2.4 Statistical analysis

Categorical data are presented as counts and percentages. Number of observations available (*n*), mean ± standard deviation (SD), median, and minimum and maximum values have been reported, when appropriate. The statistical test was conducted at a 5% significance level. The minimum sample size required for this study was 4387 with a 2‐sided 99% confidence interval, assuming that the percentage of participants with composite incidence of non‐fatal myocardial infarction, stroke, and CV death after 3 years would be 3%. It was calculated that the inclusion of approximately 6300 participants will allow estimating this percentage with a precision of at least 1%, after considering that ~30% of the participants will drop out from the study before the end of the 3 years. The eligible population includes all participants who met the inclusion/exclusion criteria for the study.

## 3 RESULTS

**FIGURE 1:**
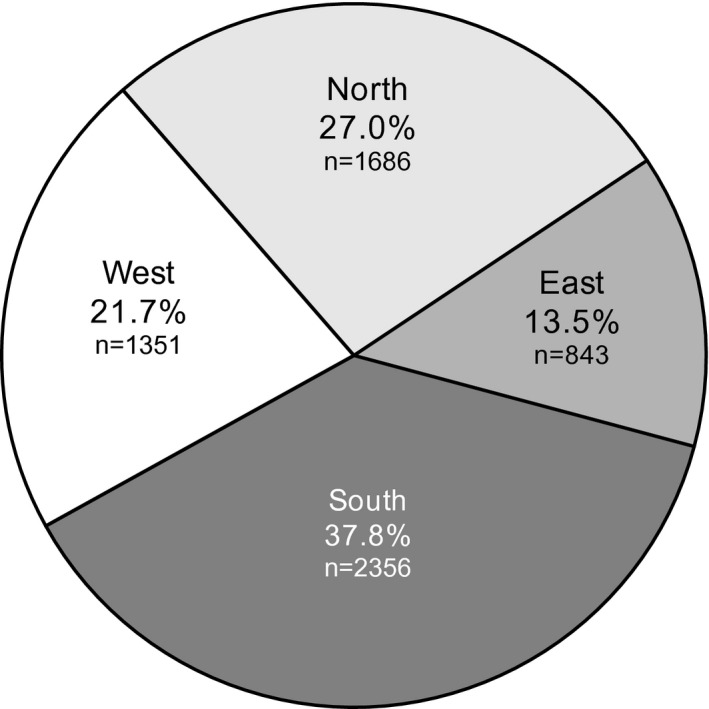
Participant recruitment status across 4 geographical regions in India, *N* = 6236. *N*, number of participants analyzed; *n*, number of participants in the region

The LANDMARC study included 382 sites across India (number of sites [participants]; East = 52 [*n* = 843], West = 81 [*n* = 1351], North = 114 [*n* = 1686], and South = 135 [*n* = 2356]; Figure 1).

### 3.1 Demographics and anthropometry

In total, 6279 participants with T2DM were recruited; of these, 6236 participants were eligible for baseline assessment (defined as the eligible population). Among the eligible population, 56.6% (*n*/*N* = 3528/6236) participants were men and 43.4% (*n*/*N* = 2708/6236) were women. Overall, the mean ± SD age was 52.1 ± 9.2 years, and 57.0% (*n*/*N* = 3553/6236) of the participants were 50–65 years old. The mean ± SD BMI was 27.2 ± 4.6 kg/m^2^, and two‐thirds (*n*/*N* = 4150/6217, 66.8%) of the participants were obese (BMI ≥ 25 kg/m^2^; Table 1).

**TABLE 1:** Demographics and clinical characteristics at baseline, *N* = 6236

Parameters	Participants
Age (years)	
Mean ± SD (years)	52.1 ± 9.2
≤30 years	61 (1.0)
31–49 years	2193 (35.2)
50–65 years	3553 (57.0)
≥66 years	429 (6.9)
Gender	
Men	3528 (56.6)
Women	2708 (43.4)
Body mass index, *n*	6217
Mean ± SD (kg/m^2^)	27.2 ± 4.6
Underweight (<18.0)	44 (0.7)
Normal (18.0–22.9)	903 (14.5)
Overweight (23.0–24.9)	1120 (18.0)
Obese (≥25.0)	4150 (66.8)
Treatment at baseline, *n*	6236
Insulin	1548 (24.8)
Insulin‐naive	4688 (75.2)
HbA1c (%), *n*	4479
Mean ± SD (%)	8.1 ± 1.6
Mean ± SD (mmol/mol)	64 ± 17
<6.5% (<48 mmol/mol)	529 (11.8)
6.5%−6.9% (48–52 mmol/mol)	593 (13.2)
<7% (<53 mmol/mol)	1122 (25.1)
7%−7.9% (53–63 mmol/mol)	1420 (31.7)
8%−8.9% (64–74 mmol/mol)	923 (20.6)
≥9% (≥75 mmol/mol)	1014 (22.6)
Fasting plasma glucose, *n*	5014
Mean ± SD (mg/dl)	142.8 ± 50.4
Mean ± SD (mmol/L)	7.9 ± 2.8
Postprandial glucose, *n*	4910
Mean ± SD (mg/dl)	205.7 ± 72.3
Mean ± SD (mmol/L)	11.4 ± 4.0
Duration of T2DM (years)	
Mean ± SD	8.6 ± 5.6
Median (range)	7.1 (2.0, 40.7)
Duration of T2DM (years), mean ± SD	
Insulin	11.3 ± 6.6
Insulin‐naive	7.7 ± 5.0

Values are presented as *n* (%) unless specified otherwise.

Abbreviations: HbA1c, glycated hemoglobin; *N*, number of participants analyzed; *n*, number of participants with non‐missing results at the visit; SD, standard deviation; T2DM, type 2 diabetes mellitus.

### 3.2 Diabetes duration and management

**FIGURE 2:**
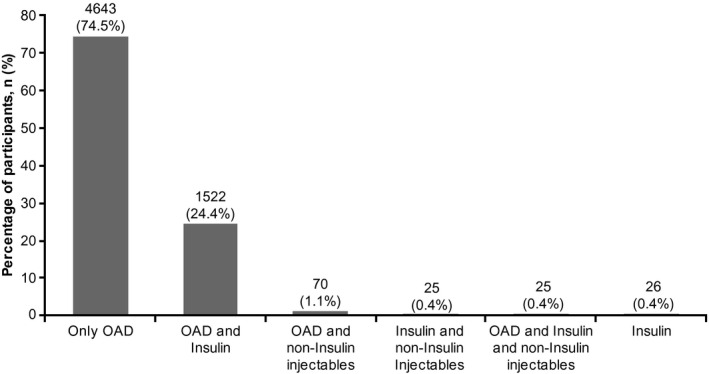
Use of oral and injectable glucose‐lowering therapies at baseline, *N* = 6236. OAD, oral anti‐diabetic drug; *N*, number of participants analyzed; *n*, number of participants with non‐missing results at the visit

**FIGURE 3:**
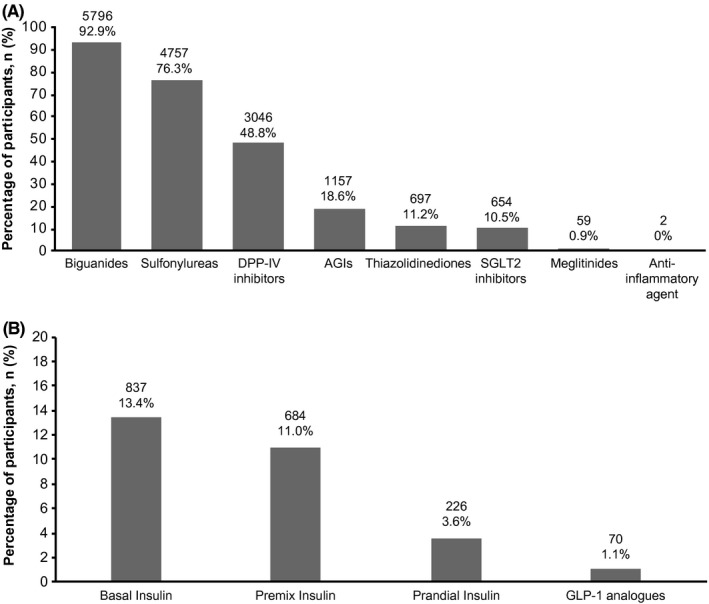
Use of oral and injectable anti‐diabetic drugs at baseline, *N* = 6236. (A) Oral anti‐diabetic drugs, (B) Injectable glucose‐lowering drugs. AGIs, alpha‐glucosidase inhibitors; DPP‐IV, dipeptidyl peptidase IV; GLP‐1, glucagon‐like peptide‐1; SGLT2, sodium glucose co‐transporter 2; *N*, number of participants analyzed; *n*, number of participants with non‐missing results at the visit: In (A), participant count for the anti‐inflammatory drug is 2 (0.0003%). Percentages are based on the total number of participants in the study (*N* = 6236). Overall, use of oral anti‐diabetic drugs was reported in 99.6% participants (*n*/*N* = 6210/ 6236). Overall, use of glucose‐lowering injectable was reported in 25.5% (*n*/*N* = 1593/6236)

At baseline, the overall mean ± SD duration of T2DM was 8.6 ± 5.6 years (median [range]: 7.1 [2.0, 40.7] years; Table 1); in most participants (*n*/*N* = 4508/6236, 72.3%), the duration of diabetes was ≤10 years (Table S1). Most participants (*n*/*N* = 4688/6236, 75.2%) were insulin‐naïve (Table 1) and were taking only oral anti‐diabetic drugs (OADs; *n*/*N* = 4643/6236, 74.5%); 25.5% (*n*/*N* = 1593/6236) were taking an injectable glucose‐lowering drug; 24.4% (*n*/*N* = 1522/6236) were taking OADs+insulin; and 0.4% (*n*/*N* = 26/6236) of participants were on insulin alone (Figures 2 and 3). The duration of diabetes in insulin‐naïve participants was shorter than that in insulin‐treated participants (7.7 ± 5.0 years vs. 11.3 ± 6.6 years; Table 1). As diabetes progressed (2–5 years to 6–10 years to >10 years), the proportion of participants on only OADs declined (from 85.1% to 76.5% to 57.4%, respectively), and the proportion of those receiving OADs+insulin increased (from 14.1% to 22.5% to 40.8%, respectively; Table S1).

**FIGURE 4:**
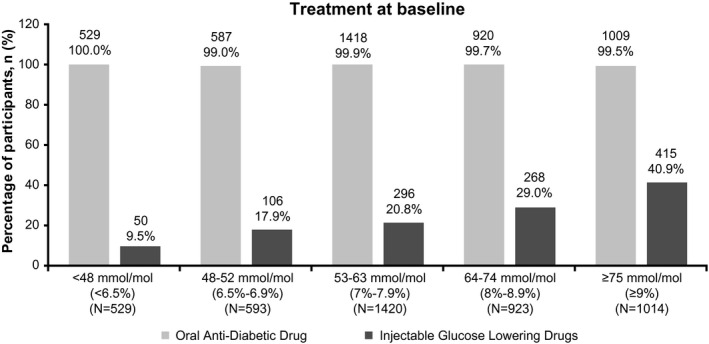
Use of oral and injectable anti‐diabetic therapies by glycemic status at baseline. HbA1c, glycated hemoglobin; *N*, number of participants analyzed; *n*, number of participants with non‐missing results at the visit. Percentages are based on the total number of participants in each subgroup stratified by glycemic status

Most participants (*n* = 2957/6236, 47.4%) were on 2 OADs, 34.0% (*n*/*N* = 2118/6236) were on 3 OADs, and 14.1% (*n*/*N* = 878/6236) were on ≥4OADs (Table S2). The most commonly noted OADs were biguanides (*n*/*N* = 5796/6236, 92.9%) and sulfonylureas (*n*/*N* = 4757/6236, 76.3%; Figure 3A). Basal insulin (*n*/*N* = 837/6236, 13.4%) and premix insulin (*n*/*N* = 684/6236, 11.0%) were the most commonly reported insulins (Figure 3B). Use of injectable glucose‐lowering drugs was also found to be greater at higher glycemic levels (Figure 4).

### 3.3 Baseline glycemic status

Overall, mean HbA1c was 64 ± 17 mmol/mol [8.1 ± 1.6%] (*n* = 4479), FPG was 142.8 ± 50.4 mg/dl [7.9 ± 2.8 mmol/L] (*n* = 5014), and PPG was 205.7 ± 72.3 mg/dl [11.4 ± 4.0 mmol/L] (*n* = 4910; Table 1). Only one‐fourth of the participants (*n*/*N* = 1122/4479, 25.1%) had HbA1c <53 mmol/mol (<7%). Among those with uncontrolled glycemia (HbA1c ≥ 53 mmol/mol, ≥7%), most (*n*/*N* = 1420/4479, 31.7%) had HbA1c 53–63 mmol/mol (7%–7.9%), whereas 20.6% (*n*/*N* = 923/4479) had HbA1c 64–74 mmol/mol (8%–8.9%) and 22.6% (*n*/*N* = 1014/4479) had HbA1c ≥ 75 mmol/mol (≥9%; Table 1).

Although suboptimal, the glycemic levels (mean HbA1c, FPG and PPG) did not vary widely across 4 geographical regions, gender, and BMI categories (Tables S3 and S4). Likewise, the mean glycemic levels were above the recommended targets across treatment subgroups (insulin‐naïve and insulin‐treated) and lower in the insulin‐naïve subgroup compared with the insulin‐treated subgroup (mean ± SD HbA1c: 62 ± 16 mmol/mol vs. 72 ± 20 mmol/mol [7.8 ± 1.5% vs. 8.7 ± 1.8%]; FPG: 138.4 ± 46.0 mg/dl vs. 156.0 ± 59.8 mg/dl [7.7 ± 2.6 mmol/L vs. 8.7 ± 3.3 mmol/L] and PPG: 198.9 ± 67.5 mg/dl vs. 226.1 ± 81.7 mg/dL [11.1 ± 3.8 mmol/L vs. 12.6 ± 4.5 mmol/L]; Table S5).

### 3.4 Diabetes complications

At baseline, the crude prevalence of diabetes‐related complications was 17.1% (*n*/*N* = 1069/6236; Table S3). Among the 1069 participants with diabetic complications, 799 (74.7%) had CV risks such as hypertension (603, 75.5%) and dyslipidemia (476, 59.6%; Table S6).

The crude prevalence of macrovascular complications (number of complications = 149) was 2.3% (*n*/*N* = 145/6236). The most commonly reported macrovascular complication was non‐fatal myocardial infarction (*n*/*N* = 74/145, 51.0%), followed by peripheral vascular disease (*n*/*N* = 45/145, 31.0%) and non‐fatal stroke (*n*/*N* = 30/145, 20.7%; Table S7). The prevalence of macrovascular complications was the highest in the Western region of India (*n*/*N* = 51/1351, 3.8%; Table S3). A higher prevalence of macrovascular complications was noted in the following: those with HbA1c ≥ 75 mmol/mol or ≥9% (*n*/*N* = 27/1014, 2.7%; Table 2), in men (*n*/*N* = 102/3528, 2.9%), and older participants (age ≥ 66 years: *n*/*N* = 24/429, 5.6%; Table S7 and S8); in participants having retinopathy (*n*/*N* = 16/141, 11.3%) or nephropathy (*n*/*N* = 17/154, 11.0%) vs. those having neuropathy (*n*/*N* = 45/737, 6.1%; Table S9) and in insulin‐treated (*n*/*N* = 71/1548, 4.6%) vs. insulin‐naïve (*n*/*N* = 74/4688, 1.6%) participants (Table S5). Interestingly, history of non‐fatal myocardial infarction was more in men than in women (*n*/*N* = 60/102, 58.8% vs. *n*/*N* = 14/43, 32.6%), whereas peripheral vascular disease was more evident in women than in men (*n*/*N* = 22/43, 51.2% vs. *n*/*N* = 23/102, 22.5%; Table S7). Macrovascular complications were present in 6.3% (*n*/*N* = 57/902) participants having microvascular complications and in 3.8% (*n*/*N* = 126/3281) participants having CV risk factors (Table S9 and S10).

**TABLE 2:** Diabetes complication and cardiovascular risk factor by glycemic status at baseline

Complications or risk factors	<48 mmol/mol (<6.5%) HbA1c (*N* = 529)	48–52 mmol/mol (6.5%‐6.9%) HbA1c (*N* = 593)	53–63 mmol/mol (7%‐7.9%) HbA1c (*N* = 1420)	64–74 mmol/mol (8%‐8.9%) HbA1c (*N* = 923)	≥75 mmol/mol (≥9%) HbA1c (*N* = 1014)	Total participants with known HbA1c (*N* = 4479)
Macrovascular						
Number of macrovascular complications, Ne	13	11	35	18	27	104
Participants with macrovascular complications	13 (2.5)	9 (1.5)	35 (2.5)	18 (2.0)	27 (2.7)	102 (2.3)
Non‐fatal myocardial infarction	7 (53.8)	6 (66.7)	21 (60.0)	11 (61.1)	9 (33.3)	54 (52.9)
Non‐fatal stroke	2 (15.4)	3 (33.3)	7 (20.0)	2 (11.1)	7 (25.9)	21 (20.6)
Cardiovascular death	0	0	0	0	0	0
Peripheral vascular disease	4 (30.8)	2 (22.2)	7 (20.0)	5 (27.8)	11 (40.7)	29 (28.4)
Microvascular						
Number of microvascular complications, Ne	75	91	211	148	197	722
Participants’ with microvascular complications	69 (13.0)	81 (13.7)	187 (13.2)	135 (14.6)	167 (16.5)	639 (14.3)
Neuropathy	55 (79.7)	59 (72.8)	152 (81.3)	115 (85.2)	127 (76.0)	508 (79.5)
Nephropathy	15 (21.7)	19 (23.5)	33 (17.6)	13 (9.6)	40 (24.0)	120 (18.8)
Retinopathy	5 (7.2)	13 (16.0)	26 (13.9)	20 (14.8)	30 (18.0)	94 (14.7)
Cardiovascular risks						
Number of cardiovascular risks, Ne	390	459	1094	641	687	3271
Participants with cardiovascular risks	300 (56.7)	335 (56.5)	773 (54.4)	476 (51.6)	501 (49.4)	2385 (53.2)
Hypertension	229 (76.3)	251 (74.9)	606 (78.4)	366 (76.9)	393 (78.4)	1845 (77.4)
Dyslipidemia	141 (47.0)	181 (54.0)	432 (55.9)	251 (52.7)	243 (48.5)	1248 (52.3)
Albuminuria	13 (4.3)	17 (5.1)	39 (5.0)	21 (4.4)	36 (7.2)	126 (5.3)
Family history of PCD	7 (2.3)	10 (3.0)	17 (2.2)	3 (0.6)	15 (3.0)	52 (2.2)

Values are presented as *n* (%) unless specified otherwise

Abbreviations: HbA1c, glycated hemoglobin; *N*, number of participants analyzed; *n*, number of participants with non‐missing results at the visit; Ne, number of events; PCD, premature coronary disease.

The crude prevalence of microvascular complications (number of microvascular complications = 1032) was 14.5% (*n*/*N* = 902/6236 participants; Table S7). The most commonly reported microvascular complication was neuropathy (*n*/*N* = 737/902, 81.7%), followed by nephropathy (*n*/*N* = 154/902, 17.1%) and retinopathy (*n*/*N* = 141/902, 15.6%; Table S7). The prevalence of microvascular complications was the highest in the Southern region of India (*n*/*N* = 434/2356, 18.4%; Table S3). A higher prevalence of microvascular complications was noted in those with HbA1c ≥ 75 mmol/mol or ≥9% (*n*/*N* = 167/1014, 16.5%; Table 2); in older participants (age ≥ 66 years: *n*/*N* = 86/429, 20.0%; Table S8); in participants having albuminuria (*n*/*N* = 97/153, 63.4%; Table S10); and in insulin‐treated (*n*/*N* = 384/1548, 24.8%) vs. insulin‐naïve (*n*/*N* = 518/4688, 11.0%) participants (Table S5). Microvascular complications were present in 39.3% (*n*/*N* = 57/145) participants with macrovascular complications and in 20.5% (*n*/*N* = 672/3281) participants with CV risk factors (Table S10 and S11).

The proportion of participants having microvascular complications increased with increasing HbA1c levels; 13.4% participants at <53 mmol/mol HbA1c (<7%), 13.2% at 53–63 mmol/mol HbA1c (7–7.9%), 14.6% at 64–74 mmol/mol HbA1c (8–8.9%), and 16.5% at ≥75 mmol/mol HbA1c (≥9%). On the contrary, the proportion of participants with macrovascular complications was almost similar across HbA1c levels; 2.0% participants at <53 mmol/mol HbA1c (<7%), 2.5% at 53–63 mmol/mol HbA1c (7–7.9%), 2.0% at 64–74 mmol/mol HbA1c (8–8.9%), and 2.7% at ≥75 mmol/mol HbA1c (≥9%; Table 2).

### 3.5 Cardiovascular risk factors

At baseline, 52.6% (*n*/*N* = 3281/6236) participants had underlying CV risks; the most prevalent CV risks were hypertension (*n*/*N* = 2566/3281, 78.2%) and dyslipidemia (*n*/*N* = 1635/3281, 49.8%). Although 41.1% (*n*/*N* = 2564/6236) participants had no CV risks, 6.3% (*n*/*N* = 391/6236) had an unknown CV risk status and none of the participants missed the screening (Table S3). The prevalence of CV risks was the lowest in North India (*n*/*N* = 687/1686, 40.7%; Table S3). Proportions of participants with CV risks were almost similar across all HbA1c categories. (Table 2) CV risks were seen more in insulin‐treated participants than in insulin‐naïve participants (*n*/*N* = 931/1548 [60.1%] vs. *n*/*N* = 2350/4688 [50.1%]; Table S5). Although CV risks were seen in nearly half of the obese (BMI ≥25 kg/m^2^; *n*/*N* = 2305/4150, 55.5%) and overweight (23.0–24.9 kg/m^2^) participants (*n*/*N* = 551/1120, 49.2%), they were also noted in half of the underweight (BMI < 18 kg/m^2^) participants (*n*/*N* = 22/44, 50.0%; Table S12). The proportion of participants with CV risks increased consistently with increase in age and was the highest in older participants (age ≥ 66 years: *n*/*N* = 297/429, 69.2%; Table S8).

## 4 DISCUSSION

This pan‐India, real‐world, longitudinal study aims to assess glycemic control and development of macro‐ and microvascular complications for a period of 3 years and explore the treatment adaptation trends in a vast sample of adults with T2DM. This article presents the demographics and clinical/medical profile of study participants at the study entry.

At baseline, only 25.1% of the study population had optimal glycemic control (HbA1c < 53 mmol/mol; <7%). This result is similar to that of the TIGHT (The Investigation of Glycosylated Haemoglobin on Therapy in Indian diabetics) study (23.4%)^15^ and the International Diabetes Management Practices Study (IDMPS): wave‐5 (2011–2012, 26.0% of participants from India had HbA1c < 53 mmol/mol or <7%) ^16^ and wave‐7 (2016, 25.2% of participants had HbA1c 53 mmol/mol or <7%).^17^ This result is also similar to that of a report from North Kerala (28.3%, HbA1c < 53 mmol/mol or <7%)^18^ and to that of a recent national diabetes registry program conducted across 200 diabetes clinics/centers in India (23.4%).^19^ Further, the 1st phase of the multicentric Indian Council of Medical Research‐India Diabetes (ICMR‐INDIAB) study conducted in 480 participants with self‐declared diabetes reports a slightly higher (31.0%) proportion of participants with glycemic control.^20^ These studies cumulatively indicate substantial and persistent prevalence of suboptimal glycemic control for over a decade in India.

In this study, duration of diabetes, intensification of treatment, and prevalence of diabetes‐related complications seemed to be linearly related. Participants with shorter duration of diabetes were mainly on only OADs, whereas those with long‐standing diabetes were on combination therapies (mainly OADs and insulin). This is consistent with the results of the TIGHT study.^15^ In real‐world settings, the guarded step‐wise approach followed to manage diabetes results in a substantial burden of the uncontrolled disease. Nearly, 14.1% participants in this study were on >3 OADs; this may indicate clinical inertia toward insulin. Early initiation of combination therapy with timely introduction of insulin can be instrumental in achieving glycemic control.^15^ Additionally, the prevalence of vascular complications and associated risk factors (hypertension and dyslipidemia) stress the importance of employing optimal early management strategies in people with T2DM. In this study, occurrence of macro‐ and microvascular complications was higher in insulin‐treated participants compared with insulin‐naïve participants, possibly owing to the shorter duration of diabetes in the latter.

At baseline, nearly 15% of the participants had a microvascular complication; the most prevalent was neuropathy, followed by nephropathy and retinopathy. The population‐based Chennai Urban Rural Epidemiology Study (CURES, *N* = 1608)^21^ reports a 25.7% prevalence for neuropathy, 17.5% for retinopathy, and 5.1% for nephropathy. The A1chieve study reports a 24.6% prevalence for neuropathy, 21.1% for renal complications, and 16.6% for eye complications in an Indian cohort with T2DM (*N* = 19346).^22^ The studies listed above, though different in study designs, underscore the substantial prevalence of diabetic complications, especially neuropathy, in India.

Long‐term diabetes‐related complications can be prevented by timely monitoring and control of glycemic parameters. Clinical guidelines highly recommend HbA1c monitoring^23^ to aid treatment decisions. Although testing HbA1c levels is common practice to diagnose diabetes, ~28% participants in LANDMARC study did not have an HbA1c record at study entry. This suggests that HbA1c is yet not monitored routinely in some clinical settings in India and that other measures of glycemic assessments such as FPG, PPG, and self‐monitoring of blood glucose are preferred to monitor glucose levels and support treatment decisions in real‐world settings.^24^

The main strength of this study is prospective real‐world evidence generation through a large number of diverse centers across India. Another advantage is the longitudinal study design, which is a powerful way to understand the patterns or trends in the progression and management of diabetes pan‐India. This study has some limitations as well. The study is observational in nature and does not mandate any study‐specific procedures, including screening for complications or CV risks, and therefore represents the real‐world scenario where there is no adjudication of the complications or risk factors. This study does not capture data on factors such as financial status, educational qualification of the participants, and access to treatment facilities that could be investigated to understand the results better. It was noted that complications in 2.9% (*n*/*N* = 183/6236) cases were reported as unknown. Hence, the prevalence of diabetes‐related complications was a crude estimate that may have undermined the actual rate. In many regions, even the simplest methods may not be feasible for screening certain complications, such as retinopathy. All laboratory values and variables reported were per standard practice at the individual sites.

## 5 CONCLUSIONS

Analysis of baseline data from LANDMARC helps understand the clinical/medical profile of study participants and underscores the extent to which suboptimal glycemic control and associated complications are prevalent in India. There is therefore a felt need for diabetes awareness and education, supported by community‐based healthcare interventions for early diagnosis as well as assessment and treatment of diabetes, its complications, and associated co‐morbidities. This is the entry‐stage data from the LANDMARC study, and further longitudinal information will add to our understanding on the management and real‐world outcomes of T2DM in India.

## CONFLICT OF INTEREST

AKD, AM, AGU, and NR received honoraria from Sanofi and other pharmaceutical companies. KMPK is on the advisory board of Sanofi and received honorarium for his talks. SJ received speaker/advisory/research grants from Abbott, Astrazeneca, Biocon, Boehringer Ingelheim, Eli Lilly, Franco Indian, Glenmark, Lupin, Marico, MSD, Novartis, Novo Nordisk, Roche, Sanofi, Serdia, Twinhealth, and Zydus. SK received honoraria/ speaker fees from Eli Lilly, Novo Nordisk, and Sanofi. HT received honoraria from MSD, Novartis, Sanofi and from other companies for advice and lectures. BS received honorarium from Aventis, Novo Nordisk, Eli Lilly, Boehringer Ingelheim (BI), and MSD. RG, AN, SM, SKM, VK, DC, VS, CT, and GC are employees of Sanofi. SC received honoraria/grants from Biocon, BI, Intas, Novartis, Sanofi, and Serdia. SKW has nothing to declare. AHZ received honoraria from Novo Nordisk, Eli Lilly, Johnson & Johnson, AstraZeneca, BI, and Sanofi.

## AUTHOR CONTRIBUTIONS

AKD, SKM, and CT were involved in the study concept and design. DC was involved in study concept and design, data analyses, and drafting the study report. All the authors participated in the interpretation of data and the writing, reviewing, and editing of the manuscript and had final responsibility for approving the published version of the manuscript. AKD is the guarantor of this work and, as such, had full access to all the data in the study and takes responsibility for the integrity of the data and the accuracy of the data analysis.

## Supporting information

Table S1‐S12

## Data Availability

Qualified researchers may request access to person‐level data and related study documents including the clinical study report, study protocol with any amendments, blank case report form, statistical analysis plan, and dataset specifications. Person‐level data will be anonymized, and study documents will be redacted to protect the privacy of trial participants. Further details on Sanofi's data sharing criteria, eligible studies, and process for requesting access can be found at https://www.clinicalstudydatarequest.com.
