# Supplementary material for: Baseline characteristics of participants in the LANDMARC trial: A 3‐year, pan‐india, prospective, longitudinal study to assess management and real‐world outcomes of diabetes mellitus
Source: Endocrinol Diabetes Metab. 2021 Feb 8;4(3):e00231. doi: 10.1002/edm2.231 (PMC8279635; doi:10.1002/edm2.231)
Supplement: Supplementary file 1 — Table S1‐S12 [file EDM2-4-e00231-s001.doc]

# Supporting information:

# Supplementary Table S1: Diabetes treatment stratified by diabetes duration at baseline

| Duration of diabetes | **2–5 years** | **6-10 years** | **>10 years** | **Total** |
| --- | --- | --- | --- | --- |
| N | 2360 | 2148 | 1728 | 6236 |
| Baseline, n (%) |  |  |  |  |
| Only on OAD | 2008 (85.1) | 1643 (76.5) | 992 (57.4) | 4643 (74.5) |
| Only on Insulin | 8 (0.3) | 5 (0.2) | 13 (0.8) | 26 (0.4) |
| On OAD and Insulin | 333 (14.1) | 484 (22.5) | 705 (40.8) | 1522 (24.4) |
| On OAD and non-Insulin injectables | 13 (0.6) | 24 (1.1) | 33 (1.9) | 70 (1.1) |
| On Insulin and non-Insulin injectables | 2 (0.1) | 8 (0.4) | 15 (0.9) | 25 (0.4) |
| On OAD and Insulin and non-insulin injectables | 2 (0.1) | 8 (0.4) | 15 (0.9) | 25 (0.4) |

OAD, oral anti-diabetic drugs

N, number of participants analysed; n, number of participants with non-missing results at the visit

# Supplementary Table S2: Proportion of participants by oral anti-diabetic drug count

| **Anti-diabetic drug count** | **Total (N=6236) n (%)** |
| --- | --- |
| 1 OAD | 257 (4.1) |
| 2 OADs | 2957 (47.4) |
| 3 OADs | 2118 (34.0) |
| 4 OADs | 752 (12.1) |
| 5 OADs | 121 (1.9) |
| >5 OADs | 5 (0.1) |

OAD, oral anti-diabetic drugs

N, number of participants analysed; n, number of participants with non-missing results at the visit

# Supplementary Table S3: Summary of glycaemic status and micro- and macro-vascular complications at baseline by region

|  |  | **East (N=843)** | **West (N=1351)** | **North (N=1686)** | **South (N=2356)** | **Total (N=6236)** |
| --- | --- | --- | --- | --- | --- | --- |
| Glycaemic status |  |  |  |  |  |  |
| HbA1c mean±SD | n | 643 | 931 | 1215 | 1690 | 4479 |
|  | % | 7.9±1.7 | 7.9±1.5 | 8.1±1.6 | 8.1±1.6 | 8.1±1.6 |
|  | mmol/mol | 63±18 | 63±16 | 65±17 | 65±18 | 64±17 |
| Fasting plasma glucose  mean±SD | n | 737 | 1087 | 1120 | 2070 | 5014 |
|  | mg/dL | 142.2±50.3 | 135.8±45.2 | 145.0±50.7 | 145.5±52.4 | 142.8±50.4 |
|  | mmol/L | 7.9±2.8 | 7.5±2.5 | 8.1±2.8 | 8.1±2.9 | 7.9±2.8 |
| Post prandial glucose mean±SD | n | 702 | 1111 | 999 | 2098 | 4910 |
|  | mg/dL | 204.0±78.2 | 192.8±68.0 | 205.0±70.9 | 213.5±72.1 | 205.7±72.3 |
|  | mmol/L | 11.3±4.3 | 10.7±3.8 | 11.4±3.9 | 11.9±4.0 | 11.4±4.0 |
|  | | | | | | |
| Diabetes complications | n | 111 | 278 | 191 | 489 | 1069 (17.1) |
| Myocardial infarction | | 3 (2.7) | 27 (9.7) | 8 (4.2) | 36 (7.4) | 74 (6.9) |
| Stroke | | 3 (2.7) | 10 (3.6) | 2 (1.0) | 15 (3.1) | 30 (2.8) |
| Peripheral vascular disease | | 0 | 17 (6.1) | 9 (4.7) | 19 (3.9) | 45 (4.2) |
| Neuropathy | | 63 (56.8) | 188 (67.6) | 117 (61.3) | 369 (75.5) | 737 (68.9) |
| Nephropathy | | 17 (15.3) | 39 (14.0) | 36 (18.8) | 62 (12.7) | 154 (14.4) |
| Retinopathy | | 13 (11.7) | 33 (11.9) | 21 (11.0) | 74 (15.1) | 141 (13.2) |
| Acute coronary syndrome | | 21 (18.9) | 25 (9.0) | 26 (13.6) | 20 (4.1) | 92 (8.6) |
| Heart failure | | 2 (1.8) | 15 (5.4) | 0 | 6 (1.2) | 23 (2.2) |
| Unstable angina | | 2 (1.8) | 21 (7.6) | 1 (0.5) | 15 (3.1) | 39 (3.6) |
| No complications | n | 718 | 1054 | 1469 | 1743 | 4984 (79.9) |
| Unknown^[1]^ |  | 14 | 19 | 26 | 124 | 183 |
|  | | | | | | |
| Macrovascular complications | Ne | 6 | 54 | 19 | 70 | 149 |
| Participants with macrovascular complications | | 6 (0.7) | 51 (3.8) | 19 (1.1) | 69 (2.9) | 145 (2.3) |
| Non-Fatal myocardial infarction | | 3 (50.0) | 27 (52.9) | 8 (42.1) | 36 (52.2) | 74 (51.0) |
| Non-fatal stroke | | 3 (50.0) | 10 (19.6) | 2 (10.5) | 15 (21.7) | 30 (20.7) |
| Cardiovascular death | | 0 | 0 | 0 | 0 | 0 |
| Peripheral vascular disease | | 0 | 17 (33.3) | 9 (47.4) | 19 (27.5) | 45 (31.0) |
| No complications | Ne | 827 | 1289 | 1657 | 2174 | 5947 |
| Unknown^[1]^, |  | 10 | 11 | 10 | 113 | 144 |
|  | | | | | | |
| Microvascular complications | Ne | 93 | 260 | 174 | 505 | 1032 |
| Participants with microvascular complications | | 87 (10.3) | 222 (16.4) | 159 (9.4) | 434 (18.4) | 902 (14.5) |
| Neuropathy | | 63 (72.4) | 188 (84.7) | 117 (73.6) | 369 (85.0) | 737 (81.7) |
| Nephropathy | | 17 (19.5) | 39 (17.6) | 36 (22.6) | 62 (14.3) | 154 (17.1) |
| Retinopathy | | 13 (14.9) | 33 (14.9) | 21 (13.2) | 74 (17.1) | 141 (15.6) |
| No complications | n | 742 | 1115 | 1513 | 1800 | 5170 |
| Unknown^[1]^ |  | 14 | 14 | 14 | 122 | 164 |
|  | | | | | | |
| Number of cardiovascular risk factors | Ne | 601 | 1112 | 843 | 1863 | 4419 |
| Participants with cardiovascular risk factors | | 448 (53.1) | 819 (60.6) | 687 (40.7) | 1327 (56.3) | 3281 (52.6) |
| Hypertension | | 358 (79.9) | 646 (78.9) | 587 (85.4) | 975 (73.5) | 2566 (78.2) |
| Dyslipidaemia | | 226 (50.4) | 418 (51.) | 216 (31.4) | 775 (58.4) | 1635 (49.8) |
| Albuminuria | | 13 (2.9) | 42 (5.1) | 30 (4.4) | 68 (5.1) | 153 (4.7) |
| Family History of PCD | | 4 (0.9) | 6 (0.7) | 10 (1.5) | 45 (3.4) | 65 (2.0) |
| No complications | n | 374 | 522 | 738 | 930 | 2564 (41.1) |
| Unknown^[1]^ |  | 21 | 10 | 261 | 99 | 391 |

^[1]^Participants who had chosen 'No' and 'Unknown' for multiple complications are counted under 'Unknown'.

HbA1c, glycated haemoglobin; SD, standard deviation; PCD, premature coronary disease

N, number of participants analysed; n, number of participants with non-missing results at the visit; Ne, number of events

Values are presented as n (%) unless specified otherwise.

**Supplementary Table S4: Summary of glycaemic status by age, gender, BMI categories, OAD count**

|  | **HbA1c** | | | **FPG** | | | **PPG** | | |
| --- | --- | --- | --- | --- | --- | --- | --- | --- | --- |
|  | **n** | **%** | **mmol/mol** | **n** | **mg/dL** | **mmol/L** | **n** | **mg/dL** | **mmol/L** |
| **Age (years): mean**±**SD** |  |  |  |  |  |  |  |  |  |
| ≤30 year | 47 | 8.0±1.6 | 64±18 | 52 | 156.8±60.1 | 8.7±3.3 | 47 | 202.7±83.4 | 11.3±4.6 |
| 31-49 years | 1600 | 8.1±1.6 | 65±18 | 1808 | 147.4±53.7 | 8.2±3.0 | 1762 | 211.1±73.2 | 11.7±4.1 |
| 50-65 years | 2557 | 8.1±1.6 | 65±17 | 2833 | 140.6±48.1 | 7.8±2.7 | 2761 | 202.9±72.1 | 11.3±4.0 |
| ≥66 years | 275 | 7.7±1.4 | 61±16 | 321 | 134.3±45.9 | 7.5±2.6 | 340 | 201.0±65.7 | 11.2±3.7 |
|  |  |  |  |  |  |  |  |  |  |
| **Gender: mean**±**SD** |  |  |  |  |  |  |  |  |  |
| Men | 2578 | 8.0±1.6 | 64±17 | 2824 | 141.6±49.5 | 7.9±2.8 | 2743 | 207.0±71.5 | 11.5±4.0 |
| Women | 1901 | 8.1±1.6 | 65±18 | 2190 | 144.4±51.4 | 8.0±2.9 | 2167 | 204.1±73.2 | 11.3±4.1 |
|  |  |  |  |  |  |  |  |  |  |
| **BMI Group kg/m^2^: mean**±**SD** |  |  |  |  |  |  |  |  |  |
| Underweight (<18.0) | 21 | 8.1±2.9 | 66±32 | 35 | 147.0±91.3 | 8.2±5.1 | 42 | 218.0±81.7 | 12.1±4.5 |
| Normal (18.0–22.9) | 632 | 8.1±1.7 | 65±19 | 726 | 144.4±50.5 | 8.0±2.8 | 717 | 211.9±76.3 | 11.8±4.2 |
| Overweight (23.0–24.9) | 791 | 8.0±1.5 | 64±16 | 907 | 140.2±47.4 | 7.8±2.6 | 891 | 202.4±68.4 | 11.2±3.8 |
| Obese (≥25.0) | 3029 | 8.0±1.6 | 64±17 | 3339 | 143.1±50.5 | 8.0±2.8 | 3253 | 205.1±72.2 | 11.4±4.0 |
|  |  |  |  |  |  |  |  |  |  |
| **Number of OADs: mean**±**SD** |  |  |  |  |  |  |  |  |  |
| 1 OAD | 170 | 8.8±2.1 | 72±23 | 193 | 158.3±62.4 | 8.8±3.5 | 189 | 227.0±85.8 | 12.6±4.8 |
| 2 OADs | 2132 | 8.0±1.6 | 64±18 | 2371 | 143.8±51.9 | 8.0±2.9 | 2328 | 205.1±74.3 | 11.4±4.1 |
| 3 OADs | 1531 | 8.1±1.5 | 65±17 | 1700 | 141.1±48.6 | 7.8±2.7 | 1661 | 205.3±70.1 | 11.4±3.9 |
| 4 OADs | 545 | 8.0±1.5 | 64±16 | 623 | 138.4±44.0 | 7.7±2.5 | 608 | 200.9±64.5 | 11.2±3.6 |
| >4 OADs | 85 | 8.2±1.4 | 66±16 | 102 | 143.1±48.5 | 8.0±2.7 | 103 | 211.0±70.1 | 11.7±3.9 |

BMI, body mass index; FPG, fasting plasma glucose; SD, standard deviation; HbA1c, glycated haemoglobin; OAD, oral anti-diabetic drugs; PPG, post prandial glucose

n, number of participants with non-missing results at the visit

# Supplementary Table S5: Glycaemic assessments and cardiovascular risks by treatment

|  |  | **Insulin treated (N=1548)** | **Insulin naive (N=4688)** | **Total (N=6236)** |
| --- | --- | --- | --- | --- |
| Glycaemic status |  |  |  |  |
| HbA1C, Mean±SD | % | 8.7±1.8 | 7.8±1.5 | 8.1±1.6 |
|  | mmol/mol | 72±20 | 62±16 | 65±17 |
| Fasting plasma glucose, Mean±SD | mg/dL | 156.0±59.8 | 138.4±46.0 | 142.8±50.4 |
|  | mmol/L | 8.7±3.3 | 7.7±2.6 | 7.9±2.8 |
| Post prandial glucose, Mean±SD | mg/dL | 226.1±81.7 | 198.9±67.5 | 205.7±72.3 |
|  | mmol/L | 12.6±4.5 | 11.1±3.8 | 11.4±4.0 |
|  |  |  |  |  |
| Total number of macrovascular complications | Ne | 74 | 75 | 149 |
| Participants with macrovascular complications |  | 71 (4.6) | 74 (1.6) | 145 (2.3) |
| Non-fatal myocardial Infarction |  | 34 (47.9) | 40 (54.1) | 74 (51.0) |
| Non-fatal Stroke |  | 16 (22.5) | 14 (18.9) | 30 (20.7) |
| Peripheral vascular disease |  | 24 (33.8) | 21 (28.4) | 45 (31.0) |
| No complications | n | 1454 | 4493 | 5947 |
| Unknown^[1]^ |  | 23 | 121 | 144 |
|  |  |  |  |  |
| Total number of microvascular complications | Ne | 478 | 554 | 1032 |
| Participants with microvascular complications |  | 384 (24.8) | 518 (11.0) | 902 (14.5) |
| Neuropathy |  | 302 (78.6) | 435 (84.0) | 737 (81.7) |
| Nephropathy |  | 82 (21.4) | 72 (13.9) | 154 (17.1) |
| Retinopathy |  | 94 (24.5) | 47 (9.1) | 141 (15.6) |
| No complications | n | 1134 | 4036 | 5170 |
| Unknown^[1]^ |  | 30 | 134 | 164 |
|  |  |  |  |  |
| Total number of cardiovascular risks | Ne | 1353 | 3066 | 4419 |
| Participants with cardiovascular risks |  | 931 (60.1) | 2350 (50.1) | 3281 (52.6) |
| Hypertension |  | 756 (81.2) | 1810 (77.0) | 2566 (78.2) |
| Dyslipidaemia |  | 503 (54.0) | 1132 (48.2) | 1635 (49.8) |
| Albuminuria |  | 71 (7.6) | 82 (3.5) | 153 (4.7) |
| Family History of PCD |  | 23 (2.5) | 42 (1.8) | 65 (2.0) |
| No complications | n | 503 | 2061 | 2564 |
| Unknown^[1]^ |  | 114 | 277 | 391 |

^[1]^Participants who had chosen 'No' and 'Unknown' for multiple complications are counted under 'Unknown'.

HbA1c, glycated haemoglobin; PCD, premature coronary disease

N, number of participants analysed; n, number of participants with non-missing results at the visit; Ne, number of events;

Values are presented as n (%) unless specified otherwise.

# Supplementary Table S6: Overview of cardiovascular risks by complications

|  |  | **MI  (N=74)** | **Stroke (N=30)** | **PVD (N=45)** | **Neuro-**  **pathy (N=737)** | **Nephro-pathy (N=154)** | **Retino-**  **pathy (N=141)** | **ACS (N=92)** | **HF (N=23)** | **UA (N=39)** | **Total (N=1069)** |
| --- | --- | --- | --- | --- | --- | --- | --- | --- | --- | --- | --- |
| Total number of CV risks | Ne | 115 | 38 | 63 | 790 | 262 | 173 | 105 | 37 | 54 | 1202 |
| Participants with CV risks |  | 67 (90.5) | 24 (80.0) | 39 (86.7) | 542 (73.5) | 130 (84.4) | 108 (76.6) | 69 (75.0) | 20 (87.0) | 33 (84.6) | 799 (74.7) |
| Hypertension |  | 56 (83.6) | 23 (95.8) | 35 (89.7) | 385 (71.0) | 106 (81.5) | 80 (74.1) | 63 (91.3) | 18 (90.0) | 28 (84.8) | 603 (75.5) |
| Dyslipidaemia |  | 46 (68.7) | 12 (50.0) | 19 (48.7) | 342 (63.1) | 85 (65.4) | 72 (66.7) | 31 (44.9) | 14 (70.0) | 21 (63.6) | 476 (59.6) |
| Albuminuria |  | 11 (16.4) | 3 (12.5) | 6 (15.4) | 49 (9.0) | 69 (53.1) | 20 (18.5) | 8 (11.6) | 5 (25.0) | 4 (12.1) | 102 (12.8) |
| F/h of PCD |  | 2 (3.0) | 0 | 3 (7.7) | 14 (2.6) | 2 (1.5) | 1 (0.9) | 3 (4.3) | 0 | 1 (3.0) | 21 (2.6) |
| No complications | n | 7 | 6 | 5 | 181 | 23 | 28 | 23 | 3 | 5 | 252 |
| Unknown^[1]^ |  | 0 | 0 | 1 | 14 | 1 | 5 | 0 | 0 | 1 | 18 |

^[1]^Participants who had chosen 'No' and 'Unknown' for multiple complications are counted under 'Unknown'.

ACS, acute coronary syndrome; CV, cardiovascular; F/h, Family history; HF, heart failure; MI, myocardial infarction; PCD, premature coronary disease; PVD, peripheral vascular disease; UA; unstable angina

N, number of participants analysed; n, number of participants with non-missing results at the visit; Ne, number of events

Values are presented as n (%) unless specified otherwise.

# Supplementary Table S7: Overview of micro- and macro-vascular complications and cardiovascular risks by gender

|  |  | **Men (N=3528)** | **Women (N=2708)** | **Total (N=6236)** |
| --- | --- | --- | --- | --- |
| Total number of macrovascular complications | Ne | 105 | 44 | 149 |
| Participants with macrovascular complications |  | 102 (2.9) | 43 (1.6) | 145 (2.3) |
| Non-fatal myocardial infarction |  | 60 (58.8) | 14 (32.6) | 74 (51.0) |
| Non-fatal stroke |  | 22 (21.6) | 8 (18.6) | 30 (20.7) |
| Cardiovascular death |  | 0 | 0 | 0 |
| Peripheral vascular disease |  | 23 (22.5) | 22 (51.2) | 45 (31.0) |
| No complications | n | 3343 | 2604 | 5947 |
| Unknown^[1]^ |  | 83 | 61 | 144 |
|  |  |  |  |  |
| Total number of microvascular complications | Ne | 587 | 445 | 1032 |
| Participants with microvascular complications |  | 507 (14.4) | 395 (14.6) | 902 (14.5) |
| Neuropathy |  | 413 (81.5) | 324 (82.0) | 737 (81.7) |
| Nephropathy |  | 88 (17.4) | 66 (16.7) | 154 (17.1) |
| Retinopathy |  | 86 (17.0) | 55 (13.9) | 141 (15.6) |
| No complications | N | 2927 | 2243 | 5170 |
| Unknown^[1]^ |  | 94 | 70 | 164 |
|  |  |  |  |  |
| Total number of CV risks | Ne | 2445 | 1974 | 4419 |
| Participants with CV risks |  | 1816 (51.5) | 1465 (54.1) | 3281 (52.6) |
| Hypertension |  | 1402 (77.2) | 1164 (79.5) | 2566 (78.2) |
| Dyslipidaemia |  | 920 (50.7) | 715 (48.8) | 1635 (49.8) |
| Albuminuria |  | 83 (4.6) | 70 (4.8) | 153 (4.7) |
| F/h of PCD |  | 40 (2.2) | 25 (1.7) | 65 (2.0) |
| No complications | N | 1480 | 1084 | 2564 |
| Unknown^[1]^ |  | 232 | 159 | 391 |
|  |  |  |  |  |

^[1]^Participants who had chosen 'No' and 'Unknown' for multiple complications are counted under 'Unknown'.

CV, cardiovascular; F/h, Family history; PCD, premature coronary disease

N, number of participants analysed; n, number of participants with non-missing results at the visit; Ne, number of events

Values are presented as n (%) unless specified otherwise.

# Supplementary Table S8: Overview of complications and cardiovascular risks by age

|  |  | **≤30 years (N=61)** | **31-49 years (N=2193)** | **50-65 years (N=3553)** | **≥66 years (N=429)** | **Total (N=6236)** |
| --- | --- | --- | --- | --- | --- | --- |
| Total number of macrovascular complications | Ne | 1 | 25 | 96 | 27 | 149 |
| Participants with macrovascular complications |  | 1 (1.6) | 25 (1.1) | 95 (2.7) | 24 (5.6) | 145 (2.3) |
| Non-fatal myocardial infarction |  | 0 | 10 (40.0) | 51 (53.7) | 13 (54.2) | 74 (51.0) |
| Non-fatal stroke |  | 1 (100.0) | 4 (16.0) | 16 (16.8) | 9 (37.5) | 30 (20.7) |
| Cardiovascular death |  | 0 | 0 | 0 | 0 | 0 |
| Peripheral vascular disease |  | 0 | 11 (44.0) | 29 (30.5) | 5 (20.8) | 45 (31.0) |
| No complications | n | 59 | 2110 | 3384 | 394 | 5947 |
| Unknown^[1]^ |  | 1 | 58 | 74 | 11 | 144 |
|  |  |  |  |  |  |  |
| Total number of microvascular complications | Ne | 0 | 269 | 660 | 103 | 1032 |
| Participants with microvascular complications |  | 0 | 242 (11.0) | 574 (16.2) | 86 (20.0) | 902 (14.5) |
| Neuropathy |  | 0 | 201 (83.1) | 466 (81.2) | 70 (81.4) | 737 (81.7) |
| Nephropathy |  | 0 | 34 (14.0) | 98 (17.1) | 22 (25.6) | 154 (17.1) |
| Retinopathy |  | 0 | 34 (14.0) | 96 (16.7) | 11 (12.8) | 141 (15.6) |
| No complications | n | 59 | 1889 | 2890 | 332 | 5170 |
| Unknown^[1]^ |  | 2 | 62 | 89 | 11 | 164 |
|  |  |  |  |  |  |  |
| Total number of CV risks | Ne | 8 | 1180 | 2809 | 422 | 4419 |
| Participants with CV risks |  | 8 (13.1) | 924 (42.1) | 2052 (57.8) | 297 (69.2) | 3281 (52.6) |
| Hypertension |  | 6 (75.0) | 622 (67.3) | 1687 (82.2) | 251 (84.5) | 2566 (78.2) |
| Dyslipidaemia |  | 2 (25.0) | 504 (54.5) | 982 (47.9) | 147 (49.5) | 1635 (49.8) |
| Albuminuria |  | 0 | 33 (3.6) | 101 (4.9) | 19 (6.4) | 153 (4.7) |
| Family history of PCD |  | 0 | 21 (2.3) | 39 (1.9) | 5 (1.7) | 65 (2.0) |
| No complications | n | 49 | 1129 | 1272 | 114 | 2564 |
| Unknown^[1]^ |  | 4 | 140 | 229 | 18 | 391 |

^[1]^Participants who had chosen 'No' and 'Unknown' for multiple complications are counted under 'Unknown'.

CV, cardiovascular; PCD, premature coronary disease

N, number of participants analysed; n, number of participants with non-missing results at the visit; Ne, number of events

Values are presented as n (%) unless specified otherwise.

# Supplementary Table S9: Summary of macrovascular complications by microvascular complications

|  |  | **Neuropathy (N=737)** | **Nephropathy (N=154)** | **Retinopathy (N=141)** | **Total (N=902)** |
| --- | --- | --- | --- | --- | --- |
| Total number of macrovascular complications | Ne | 48 | 18 | 17 | 60 |
| Participants with macrovascular complications |  | 45 (6.1) | 17 (11.0) | 16 (11.3) | 57 (6.3) |
| Non-fatal myocardial infarction |  | 20 (44.4) | 10 (58.8) | 8 (50.0) | 28 (49.1) |
| Non-fatal stroke |  | 6 (13.3) | 3 (17.6) | 3 (18.8) | 8 (14.0) |
| Cardiovascular death |  | 0 | 0 | 0 | 0 |
| Peripheral vascular disease |  | 22 (48.9) | 5 (29.4) | 6 (37.5) | 24 (42.1) |
| No complications | n | 686 | 136 | 124 | 838 |
| Unknown^[1]^ |  | 6 | 1 | 1 | 7 |

^[1]^Participants who had chosen 'No' and 'Unknown' for multiple complications are counted under 'Unknown'.

N, number of participants analysed; n, number of participants with non-missing results at the visit; Ne, number of events

Values are presented as n (%) unless specified otherwise.

# Supplementary Table S10: Overview of macro- and micro-vascular complications by cardiovascular risks

|  |  | **Hypertension (N=2566)** | **Dyslipidaemia (N=1635)** | **Albuminuria (N=153)** | **F/h of PCD (N=65)** | **Total (N=3281)** |
| --- | --- | --- | --- | --- | --- | --- |
| Total number of macrovascular complications | Ne | 114 | 77 | 20 | 5 | 130 |
| Participants with macrovascular complications |  | 110 (4.3) | 75 (4.6) | 19 (12.4) | 5 (7.7) | 126 (3.8) |
| Non-fatal myocardial infarction |  | 56 (50.9) | 46 (61.3) | 11 (57.9) | 2 (40.0) | 67 (53.2) |
| Non-fatal stroke |  | 23 (20.9) | 12 (16.0) | 3 (15.8) | 0 | 24 (19.0) |
| Peripheral vascular disease |  | 35 (31.8) | 19 (25.3) | 6 (31.6) | 3 (60.0) | 39 (31.0) |
| No complications | n | 2420 | 1556 | 133 | 59 | 3118 |
| Unknown^[1]^ |  | 36 | 4 | 1 | 1 | 37 |
|  |  |  |  |  |  |  |
| Total number of microvascular complications | Ne | 571 | 499 | 138 | 17 | 780 |
| Participants with microvascular complications |  | 491 (19.1) | 417 (25.5) | 97 (63.4) | 15 (23.1) | 672 (20.5) |
| Neuropathy |  | 385 (78.4) | 342 (82.0) | 49 (50.5) | 14 (93.3) | 542 (80.7) |
| Nephropathy |  | 106 (21.6) | 85 (20.4) | 69 (71.1) | 2 (13.3) | 130 (19.3) |
| Retinopathy |  | 80 (16.3) | 72 (17.3) | 20 (20.6) | 1 (6.7) | 108 (16.1) |
| No complications | n | 2028 | 1201 | 55 | 46 | 2556 |
| Unknown^[1]^ |  | 47 | 17 | 1 | 4 | 53 |

^[1]^Participants who had chosen 'No' and 'Unknown' for multiple complications are counted under 'Unknown'.

F/h, family history; PCD, premature coronary disease

N, number of participants analysed; n, number of participants with non-missing results at the visit; Ne, number of events

Values are presented as n (%) unless specified otherwise.

# Supplementary Table S11: Overview of microvascular complication by macrovascular complications

|  |  | **Myocardial infarction (N=74)** | **Stroke (N=30)** | **Peripheral vascular disease (N=45)** | **Total (N=145)** |
| --- | --- | --- | --- | --- | --- |
| Total number of microvascular complications | Ne | 38 | 12 | 33 | 78 |
| Participants with microvascular complications |  | 28 (37.8) | 8 (26.7) | 24 (53.3) | 57 (39.3) |
| Neuropathy |  | 20 (71.4) | 6 (75.0) | 22 (91.7) | 45 (78.9) |
| Nephropathy |  | 10 (35.7) | 3 (37.5) | 5 (20.8) | 17 (29.8) |
| Retinopathy |  | 8 (28.6) | 3 (37.5) | 6 (25.0) | 16 (28.1) |
| No complications | n | 46 | 22 | 20 | 87 |
| Unknown^[1]^ |  | 0 | 0 | 1 | 1 |

^[1]^Participants who had chosen 'No' and 'Unknown' for multiple complications are counted under 'Unknown'.

N, number of participants analysed; n, number of participants with non-missing results at the visit; Ne, number of events

Values are presented as n (%) unless specified otherwise.

# Supplementary Table S12: Overview of cardiovascular risks by body mass index (kg/m^2^)

|  |  | **Underweight (<18.0** kg/m^2^**) (N=44)** | **Normal  (18.0 – 22.9** kg/m^2^**) (N=903)** | **Overweight  (23.0 - 24.9** kg/m^2^**) (N=1120)** | **Obese  (≥25.0** kg/m^2^**) (N=4150)** | **Total (N=6217)** |
| --- | --- | --- | --- | --- | --- | --- |
| Total number of CV risks | Ne | 28 | 510 | 717 | 3161 | 4416 |
| Participants with CV risks |  | 22 (50.0) | 400 (44.3) | 551 (49.2) | 2305 (55.5) | 3278 (52.7) |
| Hypertension |  | 17 (77.3) | 290 (72.5) | 410 (74.4) | 1846 (80.1) | 2563 (78.2) |
| Dyslipidaemia |  | 11 (50.0) | 193 (48.3) | 269 (48.8) | 1162 (50.4) | 1635 (49.9) |
| Albuminuria |  | 0 | 21 (5.3) | 25 (4.5) | 107 (4.6) | 153 (4.7) |
| Family History of PCD |  | 0 | 6 (1.5) | 13 (2.4) | 46 (2.0) | 65 (2.0) |
| No complications | n | 22 | 454 | 499 | 1583 | 2558 |
| Unknown^[1]^ |  | 0 | 49 | 70 | 262 | 381 |
|  |  |  |  |  |  |  |
| Total number of microvascular complications | Ne | 3 | 169 | 204 | 654 | 1030 |
| Participants with microvascular complications |  | 3 (6.8) | 141 (15.6) | 171 (15.3) | 586 (14.1) | 901 (14.5) |
| Neuropathy |  | 3 (100.0) | 118 (83.7) | 140 (81.9) | 475 (81.1) | 736 (81.7) |
| Nephropathy |  | 0 | 21 (14.9) | 30 (17.5) | 102 (17.4) | 153 (17.0) |
| Retinopathy |  | 0 | 30 (21.3) | 34 (19.9) | 77 (13.1) | 141 (15.6) |
| No complications | n | 41 | 739 | 926 | 3457 | 5163 |
| Unknown^[1]^ |  | 0 | 23 | 23 | 107 | 153 |
|  |  |  |  |  |  |  |
| Total number of macrovascular complications | Ne | 0 | 20 | 33 | 96 | 149 |
| Participants with macrovascular complications |  | 0 | 20 (2.2) | 30 (2.7) | 95 (2.3) | 145 (2.3) |
| Non-fatal myocardial infarction |  | 0 | 10 (50.0) | 16 (53.3) | 48 (50.5) | 74 (51.0) |
| Non-fatal stroke |  | 0 | 3 (15.0) | 8 (26.7) | 19 (20.0) | 30 (20.7) |
| Cardiovascular death |  | 0 | 0 | 0 | 0 | 0 |
| Peripheral vascular disease |  | 7 (35.0) | 9 (30.0) | 29 (30.5) | 45 (31.0) | 7 (35.0) |
| No complications | n | 44 | 866 | 1067 | 3962 | 5939 |
| Unknown^[1]^ |  | 0 | 17 | 23 | 93 | 133 |
|  |  |  |  |  |  |  |

CV, cardiovascular; PCD, premature coronary disease

N, number of participants analysed; n, number of participants with non-missing results at the visit; Ne, number of events

Values are presented as n (%) unless specified otherwise.
